# A comparison of unhealthy lifestyle practices among adults with hypertension aware and unaware of their hypertensive status: results from the 2013 WHO STEPS survey in Burkina Faso

**DOI:** 10.1186/s12889-022-14026-7

**Published:** 2022-08-23

**Authors:** Jeoffray Diendéré, Jean Kaboré, William Kofi Bosu, Jérome Winbetouréfâ Somé, Franck Garanet, Pingdéwendé Victor Ouédraogo, Abdoul Aziz Savadogo, Athanase Millogo, Augustin Nawidimbasba Zeba

**Affiliations:** 1 Centre National de la Recherche Scientifique et Technologique (CNRST)/Institut de Recherche en Sciences de la Santé (IRSS), Bobo-Dioulasso, Burkina Faso; 2 Centre National de la Recherche Scientifique et Technologique (CNRST)/Institut de Recherche en Sciences de la Santé (IRSS), Ouagadougou, Burkina Faso; 3 Department of Public Health and Research, West African Health Organisation (WAHO), Bobo-Dioulasso, Burkina Faso; 4 Centre National de la Recherche Scientifique et Technologique (CNRST)/Institut de Recherche en Sciences de la Santé (IRSS) Unité de Kaya, Kaya, Burkina Faso; 5 Université Nazi Boni/Institut Supérieur des Sciences de la Santé (INSSA), Bobo-Dioulasso, Burkina Faso; 6 Université Joseph Ki-Zerbo/Centre Hospitalier Universitaire Sourô Sanou, Bobo-Dioulasso, Burkina Faso

**Keywords:** Prevalence, Unhealthy lifestyles, Undiagnosed hypertension, Awareness, WHO STEPS, Burkina Faso

## Abstract

**Background:**

We compared the prevalence of unhealthy lifestyle factors between the hypertensive adults who were aware and unaware of their hypertensive status and assessed the factors associated with being aware of one’s hypertension among adults in Burkina Faso.

**Methods:**

We conducted a secondary analysis of data from the World Health Organization Stepwise approach to surveillance survey conducted in 2013 in Burkina Faso. Lifestyle factors analysed were fruits and vegetables (FV) consumption, tooth cleaning, alcohol and tobacco use, body mass index and physical activity.

**Results:**

Among 774 adults living with hypertension, 84.9% (95% CI: 82.2–87.3) were unaware of their hypertensive status. The frequencies of unhealthy lifestyle practices in those aware vs. unaware were respectively: 92.3% vs. 96.3%, *p* = 0.07 for not eating, at least, five FV servings daily; 63.2% vs. 70.5%, *p* = 0.12 for not cleaning the teeth at least twice a day; 35.9% vs. 42.3%, *p* = 0.19 for tobacco and/or alcohol use; 53.9% vs. 25.4%, *p* = 0.0001 for overweight/obesity and 17.1% vs, 10.3%, *p* = 0.04 for physical inactivity. In logistic regression analysis, older age, primary or higher education, being overweight/obese [adjusted odds ratio (aOR) = 3.2; *p* < 0.0001], intake of adequate FV servings daily (aOR = 2.9; *p* = 0.023) and non-use of alcohol and tobacco (aOR = 0.6; *p* = 0.028) were associated with being aware of one’s hypertensive status.

**Conclusion:**

Undiagnosed hypertension was very high among Burkinabè adults living with hypertension. Those aware of their hypertension diagnosis did not necessarily practise healthier lifestyles than those not previously aware of their hypertension. Current control programmes should aim to improve hypertension awareness and promote risk reduction behaviour.

## Background

In 2019, an estimated that 1.28 billon people aged 30–79 years worldwide lived with hypertension of whom 82% lived in low- and middle-income countries (LMICs) [1]. Over the long term, hypertension leads to increased complications such as heart disease, stroke, kidney failure, disability, and premature mortality [2]. The challenge in reducing the burden of cardiovascular diseases, particularly in LMICs, includes efforts to improve diagnosis and ensure adequate management or treatment for persons living with hypertension [3]. Undiagnosed hypertension and inadequate treatment hinder the effective control of hypertension. Effective strategies for non-pharmacological hypertension management include the adherence to healthy lifestyle and dietary behaviours such as avoidance of tobacco or harmful use of alcohol, weight control, physical activity, fruit and/or vegetables (FV) consumption, as well as oral hygiene [4–7].

There is some evidence to suggest that persons living with hypertension who are aware of their condition or have knowledge about the disease may practise healthier lifestyles than those who do not [8]. We, therefore, presumed that Burkinabè adults who were aware of their hypertensive status would have healthier lifestyle measures than those who were not. Using data from the first World Health Organization (WHO) stepwise approach to surveillance (STEPS) survey in Burkina Faso, we compared the distribution of lifestyle factors between the adults living with hypertension who were aware and unaware of their hypertensive status. We also assessed the factors associated with being aware of one’s hypertensive status.

## Methods

### Description of the Burkina Faso STEPS survey

The WHO STEPS surveys use a standardized tool for data collection which includes specific sections on behavioural risk factors (tobacco and alcohol consumption, oral hygiene practices, FV intake, physical activity); anthropometric [body mass index (BMI)] and blood pressure measurements; and blood biochemistry [9].

The first WHO STEPS survey in Burkina Faso, conducted from 3 September to 24 October 2013, was nationally-representative and covered all the country’s 13 administrative regions. It involved interviews on behavioural or lifestyle factors as well as anthropometric and blood pressure measurements [9]. As the detailed methodology including sample size calculation, sampling procedure and blood pressure measurements have been previously published [10, 11], only a brief description will be presented for this secondary data analysis study.

The survey enrolled adults aged 25–64 years, based on a calculated sample size, large enough to allow sub-group comparisons. The estimated sample size, based on an assumed prevalence of hypertension of 29.4, 5% precision, a design effect of 1.5 and 20% non-response, was 4785, and was rounded up to 4800. The sample was weighted by sex, age group, and rural/urban residence.

A stratified three-stage cluster proportional to the size sampling was used to select participants. The sample was stratified to provide adequate representation of both rural and urban residence. An excel spread sheet was used to draw households from each selected cluster. One individual aged 25–64 years was randomly selected from each household using the Kish method [12]. Face-to-face interviews were conducted in a language spoken by the participant and data captured using personal digital assistants pre-loaded with eSTEPS software.

Blood pressure was measured on the right arm with an electronic blood pressure monitor (OMRON HEM-705, Tokyo, Japan) with the patient seated upright with the legs uncrossed. The blood pressure was taken three times at five-minute intervals after the participant had initially rested for 15 minutes. The mean of the three blood pressure readings was used in the analysis of hypertension.

All methods were carried out in accordance with relevant guidelines and regulations. The protocol of the primary STEPS survey was approved by the Ethics Committee for Health Research of the Ministry of Health of Burkina Faso (deliberation No: 2012–12,092; December 05, 2012). Written informed consent was systematically obtained from each participant in the STEPS survey.

As the current study did not involve human subjects, no ethical approval was necessary. The database for this secondary analysis, is freely available from the Ministry of Health of Burkina Faso on request. As the study authors work with a national research health institute that is affiliated to the Ministry of Health of Burkina Faso, there was no difficulty with access to the database.

### Study variables

Sociodemographic data collected included living environment, sex, age, marital status, education level and occupation. Self-reported data on the modifiable lifestyle factors that were considered in the secondary data analysis were alcohol and/or tobacco use, oral hygiene practices, FV consumption, and physical inactivity. The anthropometric measurements were weight, height, as well as blood pressure.

Current alcohol consumption was defined as alcohol intake in the past 1 month while current tobacco use was defined as ever use of smoked or smokeless tobacco in the past 12 months [9, 13, 14]. The oral hygiene practices were categorized based on the frequency of cleaning teeth per day, with, at least twice daily cleaning being recommended [15]. Daily FV intake was derived from the number of servings of FV consumed per day during a typical week. Five or more daily FV servings is recommended [16]. Physical activity was investigated via the amount of time being physically active in three domains; transport, at work and during leisure time and participants were asked about the frequency, intensity and duration of their work-, travel- and leisure-related physical activity (vigorous or moderate), in a typical week [17]. We considered participants who reported no vigorous- or low- physical activity during a typical week as being physically-inactive. Body mass index (BMI), calculated as a subject’s weight divided by height^2^, in kg/m^2^, was characterized as underweight (BMI < 18.5 kg/m^2^), normal (BMI = 18.5–24.9 kg/m^2^) overweight (BMI = 25–29.9 kg/m^2^) or obesity (BMI ≥30 kg/m^2^) states [18].

We defined persons living with hypertension as those with higher than or equal to 140 mmHg and/or diastolic blood pressure higher than or equal to 90 mmHg or those who reported current antihypertensive therapy use [1, 10]. Our outcome of interest for the secondary data analysis was the awareness of hypertension defined as participants with hypertension who reported having been told by a doctor or a health professional as having raised blood pressure or hypertension.

### Statistical analyses

We used StataCorp Stata Statistical Software for Windows (Version 14.0, College Station, Texas, US) to analyse the data. The continuous variables were expressed as the means ± standard deviations, while the categorical variables were expressed as percentages (%) with 95% confidence intervals (CIs). Student’s t test was used to compare continuous variables, and the chi-square or the Fishers exact tests were used to compare categorical variables.

In the stepwise logistic regression models, we dichotomized the outcome variable as being aware or unaware of one’s hypertension status. The lifestyle factors were the explanatory variables, with adjustment on sociodemographic factors (sex, age, urban-rural residence, marital status, education and occupation). The Hosmer-Lemeshow test was performed to determine the goodness-of-fit of the logistic regression models. Except for the Hosmer-Lemeshow test, for all analyses, a *p*-value below 0.05 was considered statistically significant.

## Results

Of the sample of 4800 individuals enrolled in the primary study, 105 were not eligible; 10 had invalid data on sociodemographic variables. For our secondary data analysis, the numbers with missing or implausible data on lifestyle factors were as follows: 1 for tobacco, 6 for oral hygiene practice; 279 for FV intake, 205 for BMI, and 7 for blood pressure. After excluding these individuals and 3413 normotensive individuals, 774 individuals with hypertension and complete data were included in our analysis.

The mean age of these 774 persons was 43.8 ± 11.6 years. They were predominantly male (53.9%), rural residents (71.6%), illiterates (77.5%), or engaged in an occupation without formal income (92.0%) (Table 1).

**Table 1:** Sociodemographic characteristics of overall hypertensive adults, according to the awareness of the diagnostic status

	Overall hypertensive adults			Undiagnosed hypertensive adults			Diagnosed hypertensive adults			
	*N* = 774			657			*N* = 117			
	n	%	95% CI	n	%	95% CI	n	%	95% CI	P
Residence										0.0001
- Rural area	554	71.6	68.3–74.7	494	75.2	71.7–78.4	60	51.3	41.9–60.6	
- Urban area	220	28.4	25.3–31.7	163	24.8	21.6–28.3	57	48.7	39.4–58.1	
Sex										0.069
- Male	417	53.9	50.3–57.4	363	55.2	51.4–59.1	54	46.1	36.9–55.6	
- Female	357	46.1	42.6–49.7	294	44.8	40.9–48.6	63	53.9	44.4–63.1	
Age range (years)										0.0001
- 25–34	205	26.5	23.4–29.7	190	28.9	25.5–32.6	15	12.8	7.4–20.3	
- 35–44	193	26.0	21.9–28.1	174	26.5	23.1–30.0	19	16.2	10.1–24.2	
- 44–54	197	25.4	22.4–28.7	161	24.5	21.3–28.0	36	30.8	22.6–40.0	
- 55–64	179	23.1	20.2–26.3	132	20.1	17.1–23.4	47	40.2	31.2–49.6	
Marital status										0.73
- Married/cohabitating	650	84.0	81.2–86.5	553	84.2	81.2–86.9	97	82.9	74.8–89.2	
- Single	124	16.0	13.5–18.8	104	15.8	13.1–18.8	20	17.1	10.8–25.2	
Occupation										0.009
- Employees with formal income^a^	62	8.0	6.2–10.2	45	6.9	5.0–9.1	17	14.5	8.7–22.2	
- Others ^b^	712	92.0	89.8–93.8	612	93.1	90.9–95.0	100	85.5	77.8–91.3	
Education level										0.0001
- No formal education	584	75.5	72.3–78.4	519	79.0	75.7–82.1	65	55.6	46.1–64.7	
- Primary school	111	14.3	11.9–17.0	89	13.5	11.0–16.4	22	18.8	12.2–27.1	
- Secondary or higher	79	10.2	8.2–12.6	49	7.5	5.6–9.7	30	25.6	18.0–34.5	

^a^Workers with formal monthly salary in the public or private sectors; ^b^ Others: Self-employed, house maker, jobless, students); *CI* Confidence interval at 95%

Overall, 84.9% (95% CI: 82.2–87.3) of them were not aware of their hypertensive status (Table 2). About 43% of persons living with hypertension had recently used alcohol or tobacco or both, 69.4% did not clean their teeth frequently, 11.4% were physically inactive, 95.7% consumed inadequate FV servings and 29.7% were overweight or obese.

**Table 2:** Lifestyle behaviours among overall hypertensive adults, according to the awareness towards the hypertension status

Variable	Overall hypertensive adults			Undiagnosed hypertensive adults			Diagnosed hypertensive adults			
	*N* = 774			*N* = 657			*N* = 117			
	n	%	95% CI	n	%	95% CI	n	%	95% CI	P
Alcohol and/or tobacco use										0.027
- Used neither alcohol nor tobacco	454	58.7	55.1–62.2	379	57.7	53.8–61.5	75	64.1	54.7–72.8	
- Used either alcohol or tobacco	244	31.5	28.3–34.9	206	31.3	27.8–35.1	38	32.5	24.1–41.8	
- Used both alcohol and tobacco	76	9.8	7.8–12.1	72	11.0	8.7–13.6	4	3.4	0.9–8.5	
Oral hygiene practices: tooth cleaning frequency										0.12
- Did not clean the teeth at least twice a day	537	69.4	66.0–72.6	463	70.5	66.8–73.9	74	63.2	53.8–72.0	
- Cleaning the teeth at least twice a day	237	30.6	27.4–34.0	194	29.5	26.1–33.2	43	36.8	28.0–46.2	
Physical lifestyle (frequency of physical activity per week)										0.04
- Physically active	686	88.6	86.2–90.8	589	89.7	87.1–91.9	97	82.9	74.8–89.2	
- Physically inactive	88	11.4	9.2–13.8	68	10.3	8.1–12.9	20	17.1	10.8–25.2	
Fruits and vegetables consumption										0.076
- Inadequate FV intake	741	95.7	94.1–97.0	633	96.3	94.6–97.6	108	92.3	85.9–96.4	
- Adequate FV intake	33	4.3	3.0–5.9	24	3.7	2.4–5.4	9	7.7	3.6–14.1	
Body mass index categories										0.0001
- Underweight	76	9.8	7.8–12.1	69	10.5	8.3–13.1	7	6.0	2.4–11.9	
- Normal weight	468	60.5	56.9–63.9	421	64.1	60.3–67.8	47	40.2	31.2–49.6	
- Overweight	151	19.5	16.8–22.5	117	17.8	15.0–21.0	34	29.0	21.0–38.2	
- Obese	79	10.2	8.2–12.6	50	7.6	5.7–9.9	29	24.8	17.3–33.6	

The differences in the lifestyles between those aware and unaware of their hypertension with respect to alcohol or tobacco use, physical activity and BMI were statistically significant (Table 2). Those who were aware that they had hypertension were more likely than those with undiagnosed hypertension to have abstained from tobacco or alcohol (64.1% versus 57.7%, *p* = 0.027). They were, however, less likely to be physically active (82.9% versus 89.7%, *p* = 0.04) and more than twice as likely to be overweight or obese (53.8% versus 25.4%, *p* < 0.001). The differences in oral hygiene practices and daily FV intake were not statistically significant.

In multivariable analysis, participants with hypertension who were older, had primary education or higher, were overweight/obese, were currently using and had recently used alcohol/tobacco and those who consumed adequate FV servings were significantly more likely to report having been previously diagnosed as having hypertension (Table 3). The strongest predictors of being aware of one’s hypertension were older age, educational level and BMI status. Participants aged 55–64 years old were 7.2 times as likely as those aged 25–34 years to be aware of their hypertension. Those with secondary or higher education were 5.6 times as those with no formal education to be aware of their hypertension. Participants who were overweight/obese were 3.2 times as those with normal BMI to be aware of their hypertensive status. In contrast, marital status, occupation, physical activity and frequency of teeth cleaning were not significantly associated with awareness of one’s hypertension status. Regarding the goodness-of-fit test for this logistic regression model, the Hosmer-Lemeshow chi-square test yielded a *p*-value over 0.05.

**Table 3:** Associated factors with being warrened of its hypertension status, among hypertensive adults in Burkina Faso (*n* = 744)

Variables	Univariable analysis			Multivariable analysis		
	cOR	95% CI	*p*-value	aOR	95% CI	*p*-value
Residency: Urban vs rural (Ref)	2.9	1.9–4.3	0.0001	1.4	0.8–2.3	0.24
Gender: Women vs men (Ref)	1.4	> 0.9–2.1	0.07	1.4	0.9–2.2	0.15
Age range						
- 25–34	1			1		
- 35–44	1.4	0.7–2.8	0.37	1.4	0.7–3.0	0.37
- 45–54	2.8	1.5–5.4	0.001	3.8	1.9–7.6	0.0001
- 55–64	4.5	2.4–8.4	0.0001	7.2	3.6–14.2	0.0001
Marital status: Married/cohabitating vs singles (Ref)	0.9	0.5–1.5	0.73	1.3	0.7–2.3	0.44
Occupation: Employees with formal salary vs others (Ref)	2.3	1.3–4.2	0.006	0.7	0.3–1.7	0.46
Educational level						
- No formal education	1			1		
- Primary school	2.0	1.2–3.4	0.01	2.4	1.3–4.4	0.004
- Secondary or more	4.9	2.9–8.2	0.0001	5.6	3.1–10.1	0.0001
Physical activity: Inactive vs active (Ref)	1.8	1.1–3.1	0.04	0.9	0.5–1.9	0.99
Teeth cleaning at least twice a day: Yes, vs no (ref)	1.4	0.9–2.1	0.12	0.9	0.6–1.5	0.81
BMI categories						
- Normal	1			1		
- Underweight	0.9	0.4–2.1	0.82	0.7	0.3–1.8	0.51
- Overweight/obesity	3.4	2.2–5.1	0.0001	3.2	2.0–5.1	0.0001
Recent or current use of alcohol or tobacco						
- Did not use alcohol or tobacco	1			1		
- Used alcohol and/or tobacco	0.8	0.5–1.1	0.19	0.6	0.4–0.9	0.028
Adequate FV consumption: Yes vs no (Ref)	2.2	> 0.9–4.9	0.052	2.8	1.2–6.7	0.023

The goodness-of-fit test of this logistic regression reported the chi^2^ of Hosmer-Lemeshow at 8 degrees of freedom of 4.028, with the *p*-value of 0.86

## Discussion

Our study revealed some important findings. First, we found that undiagnosed hypertension was very high (85%) among Burkinabè adults. Our finding is similar to that in Tanzania (90%) [19] and Cameroon (81%) [20] but higher that the reported levels of 78% in Angola [21], 76% in Guinea [22], 71% in Kenya [23], 65% in Ghana [24] and 60% in Nigeria [25]. The high level of undiagnosed hypertension could pose a high risk for cardiovascular complications. For example, in Nigeria, half of all acute stroke cases presented with undiagnosed hypertension [26]. More recently, the proportion of stroke due to undiagnosed hypertension in low-income countries (15.9%) was found to be about three times as high as that in high-income countries (5.6%) [27]. In acute medical care in Burkina Faso, a high prevalence of hypertension has been reported among patients with cardiocerebrovascular events: in 58%; 66%; 76 and 86% of patients with acute coronary syndromes [28] cardioembolic disorders [29] stroke [30] and non-valvular atrial fibrillation [31] respectively. Educational programs are, therefore needed to increase awareness and practice of healthier lifestyles and thereby reduce the risk of hypertension and cardiovascular diseases [32].

Secondly, we found that the pattern of modifiable unhealthy lifestyle practices between adults living with hypertension in Burkina Faso who aware and unaware of their hypertensive status was mixed. Awareness of hypertension among Burkinabe adults did not necessarily translate into healthier lifestyles. This has also been the experience elsewhere where greater awareness of hypertension did not always translate into better treatment and control of hypertension [33] or healthier lifestyles [34]. Those aware of their hypertension being more likely to be non-current or recent users of alcohol or tobacco, consumers of adequate FV servings daily but then significantly more likely to be overweight/obese.

Our findings of healthier smoking and alcohol use habits among adults aware of their hypertension agree with those of others. Participants who aware of their hypertension were more likely to reduce their alcohol consumption in Korea [8] and to accept smoking cessation in Nigeria [35]. Similarly, undiagnosed adults with hypertension in Ghana, were more likely to be current users of alcohol or tobacco [32]. Hence, improving the diagnosis of hypertension through measures such as opportunistic screening at health facilities and in communities to identify asymptomatic persons with hypertension and counselling them on harmful effects of tobacco and alcohol use are needed.

As in our study, overweight/obesity was associated with a lower risk of undiagnosed hypertension in six LMICs [36]. It is gratifying that those overweight/obese, a known risk factor for hypertension, were also more likely to be aware of their hypertension. Overweight and obese may have co-morbidities that increase their encounters with healthcare professionals and their opportunity to learn about their hypertensive status.

Thirdly, we found that a very low frequency of adequate FV intake. The low FV intake reflects the general low FV consumption ranging from 79 to 96% in West African countries [37]. FV consumption is considered to protect against hypertension [38] and stroke [39]. There is, therefore, the need for systematic dietary programmes to improve FV intake from childhood. The challenge is the ingrained cultural taste and dietary preferences among Africans, even when the food habit is known to be unhealthy [40].

Being aware of one’s hypertension diagnosis did not appear to influence physical activity or oral hygiene practices among Burkinabe adults. A similar finding has been reported in Ghana [32] as well as in Sudan where regular exercise was found to be the most challenging lifestyle change for hypertensive subjects [41]. Low adherence rates for either weight control or exercise have been commonly reported in individuals with hypertension [8] resulting in uncontrolled hypertension [42].

Healthy oral hygiene practices including frequent tooth brushing improves dyslipidaemia, particularly high-density lipoprotein cholesterol and triglyceride levels [43], and may help in the control of hypertension [44]. Choi et al. [45] demonstrated that systolic blood pressure levels progressively decreased as the frequency of toothbrushing increased. Nonetheless, there is insufficient knowledge about adequate management of patients with hypertension in dental practice [46]. Preventive dental interventions could be used to reduce the development of hypertension [7]. Awareness programs to promote oral health may have benefits on blood pressure level in general population [47].

The strengths of our study include the large representative sample and the use of standard definitions of variables. Our study also has some limitations. First, there was a high number of missing data for certain lifestyle variables. Secondly, awareness was self-reported and could not be independently verified. Thirdly, there were several variables such as salt intake [8], psychological stress and sleep quality [48] for which data were not available and so could not be included in the regression model. Fourthly, there should be caution in the interpretation of our findings due to possible reverse causality in a cross-sectional design. For example, it is not clear whether FV intake preceded or was as a result of hypertension. Finally, the level undiagnosed hypertension in 2013 may not reflect the current situation although it provides a relevant baseline against which future national surveys may be compared. A second national STEPS survey in Burkina Faso has recently been completed with analysis pending.

## Conclusion

The prevalence of undiagnosed hypertension is very high among Burkinabè adults. Whereas most participants were physically active, most did not consume adequate amounts of fruits and vegetables daily. Participants living with hypertension who reported being aware of their condition did not necessarily practise healthier lifestyles than those who had not been previously diagnosed. Further analysis is required to assess how awareness of hypertension relates to the control of hypertension. Current educational programmes for hypertension control should be intensified, starting from childhood with the aim of improving awareness and adoption of healthier lifestyles from an early stage.

## Data Availability

The database of the STEPS survey used for this secondary analysis is available at the Ministry of Health of Burkina Faso and can be requested from bicababrico78@gmail.com.

## Abbreviations

aOR: Adjusted odds ratios
BMI: Body mass index
CI: Confidence interval
cOR: Crude odds ratio
FV: Fruits and/or vegetables
kg/m^2^: Kilogramme per square metre
LMICs: Low and middle-income countries
OR: Odds ratio
STEPS: Stepwise approach to surveillance
US: United States
WHO: World Health Organization
